# Calculation model of the equivalent spiral shear stress of conditioned sand

**DOI:** 10.1371/journal.pone.0212923

**Published:** 2019-03-13

**Authors:** Xingchun Li, Nanlin Tan

**Affiliations:** 1 Department of Mechanical Engineering, School of Mechanical, Electronic and Control Engineering, Beijing Jiaotong University, Beijing, China; 2 Department of Intelligent Manufacturing, Wuyi University, Jiangmen, Guangdong, China; China University of Mining and Technology, CHINA

## Abstract

Soil is the medium that balances the soil and water pressure on the excavation surface of an earth pressure balance (EPB) shield machine, and the soil flow plasticity directly affects the working performance of the shield machine. The spiral shear stress is the main determinant of the soil flow plasticity. During the progress of the EPB shield screw conveyor normal operation, the shear effects between the muck in the spiral tube and the screw shaft, as well as the flight, are different from the shear action, such as the direct shear or the vane shear. The direct or vane shear test is now widely used to evaluate soil flow plasticity. To better investigate the shear properties between the soil and the shield conveyor casing, a model screw conveyor was developed. Based on the analysis of the stress conditions and the movement of the conditioned soil plug, the theoretical calculation model of the equivalent helical shear stress of the EPB shield screw conveyor was deduced based on the steady-state equilibrium conditions of moment of momentum. The conditioned standard sand was used in the indoor tests. The results of the indoor test of the model machine verified the rationality and effectiveness of the theoretical model. The model provides an important theoretical basis and references for the design and optimization of soil conditioning during shield tunneling.

## Introduction

Research on the static and dynamic characteristics of the interface between muck and an earth pressure balance (EPB) shield screw conveyor structure is concerned with frontier issues, such as nonlinearity, large creep and discontinuity. It is currently one of the key research subjects related to the physical and mechanical properties of conditioned soil. The model machine test is a basic method that studies the mechanical properties of the interface between muck and the EPB structure. For example, the main factors affecting the mechanical properties of the contact surface and basic laws have been obtained via experimental studies, and a constitutive model describing the mechanical properties of the contact surface has been established by Zhang and Min [1].

Under the influence of a dynamic load from the working chamber pressure and rotation of the shield screw conveyor, the conditioned soil in the spiral groove is subjected to additional stress in the pure shear state and in the spiral rotation of the principal stress axis. The direct or vane shear test can’t effectively model this type of stress environment. In this study, the shield screw model machine simulates the stress state of the muck during normal shield tunneling and then the shear stress and creep characteristics between the conditioned soil and the spiral structure in the construction environment are analyzed to guide and solve soil conditioning during the construction process. The screw conveyor model machine performs the spiral shear test on the conditioned soil under the action of the soil pressure and the rotating spiral thrust, where the loading condition is the same as that of the muck in the spiral groove of the construction site.

In light of the importance of muck properties for spewing control of the shield screw conveyors and regulation of the working chamber pressure, the addition of conditioning agents for soil improvement is an effective technical means that has been confirmed by many engineering practices. There are three main types of commonly used additives: foaming agents, bentonite slurry and macromolecular polymers. Scholars have conducted extensive research on the mechanical properties of improved muck.

The slump test, which determines the workability of concrete, is widely used to evaluate the flow characteristics of improved muck. Many researchers have reported results in this area [2–8]. The slump test is simple and economical, and the test results generally reflect the flow characteristics of the muck. Williamson et al. [3]and Jancsecz et al. [4] studied the improvement effect of foam on sand, suggesting a reasonable slump range of 200–250 mm. The improvement effect of pebble stratum with a foam and bentonite slurry was indicated to be between 150-200mm by Jiang et al. [9]. Gharahbagh et al. [10] combined soil conditioning with the tool wear test, suggesting a reasonable range of slump values of 100-250mm.

**Fig 1:**
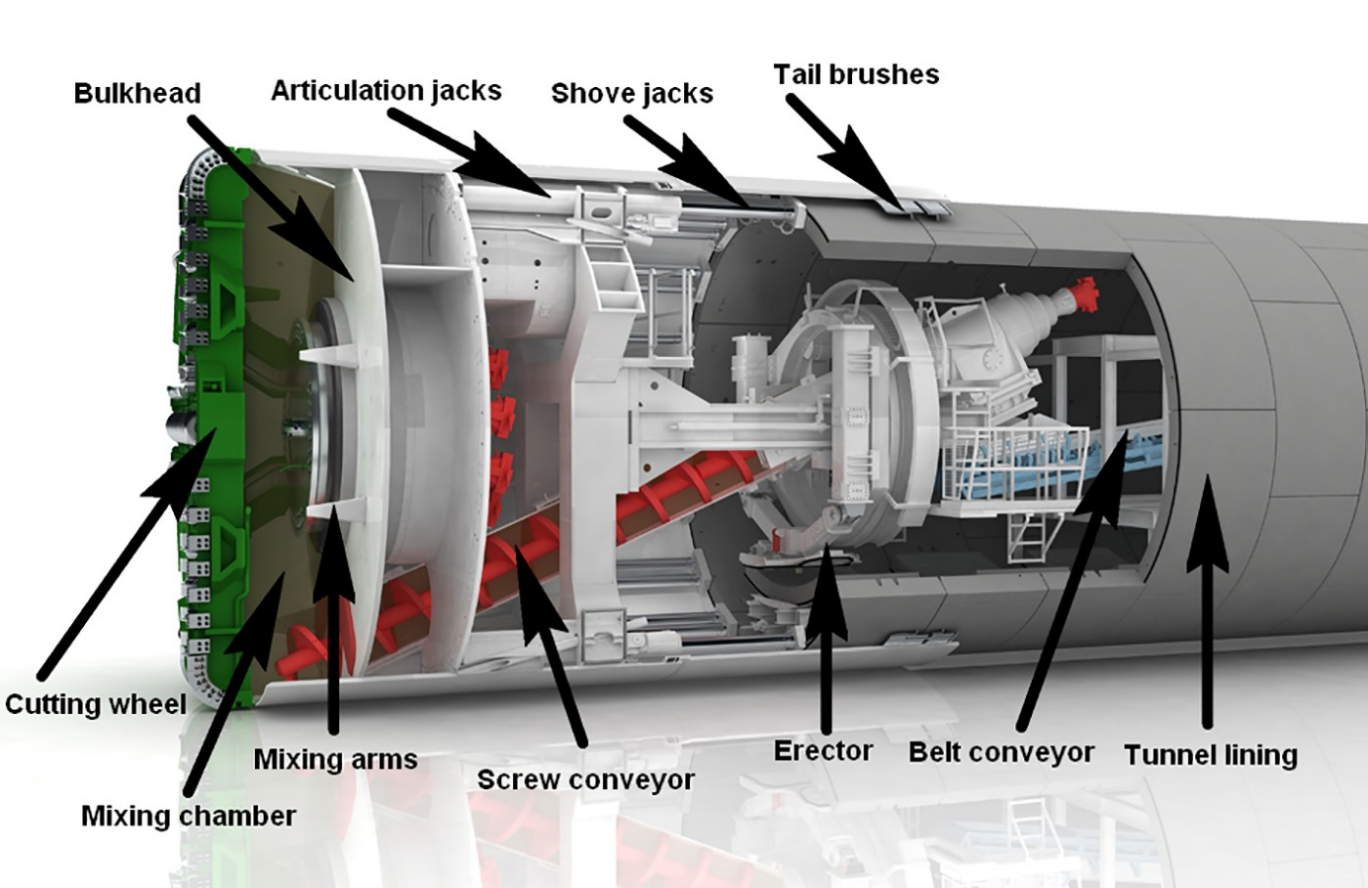
Screw conveyor in the EPB shield machine (from http://cn.herrenknecht.com/cn).

From the research results of the above studies, we can see that the slump used to measure the flow characteristics of muck is an important indicator of the conditioning effect. However, shear stress is the main influencing factor of the slump of conditioned soil. Therefore, it is necessary to study the calculation method of the spiral shear stress of conditioned soil. The shield screw conveyor is installed between the bottom of the front shield and the erector at the tail of the shield, wherein the midline rises at a certain inclination angle. When the screw conveyor is normally drained, the shaft and the spiral flight, which extend into the working chamber, are rotated by a hydraulic motor; moreover, the muck is transported and raised along a certain spiral angle under the joint action of the spiral and the outer shell and is discharged to the soil outlet. The EPB shield screw conveyor is shown in Fig 1. The helical shear stress occurs between the muck and the spiral structure. The friction and wear problems between the muck and the spiral structure and the particularity of the spiral structure make it difficult to directly measure the helical shear stress.

To better understand the ability of muck to transfer pressure and to solve the problem of homogeneous flow of muck in the screw conveyor machine, some scholars have used the spiral extruder model machine to study the flow characteristics of the muck (for example, Akimasa et al. [11]; Bezuijen et al. [12]; Bezuijen and Schaminee [13]; Merritt [14]; Bezuijen et al. [15]; Merritt and Mair [16]; Duarte [17]; Peila et al. [18]; Vinai et al. [19]).

In recent years, direct shears (Ehsan and Adam [20]; Psomas [21]; Houlsby and Psomas [22]) or modified vane shears (Zumstegt et al. [23]; Messerklinger et al. [24]; Zumsteg et al. [25]; Mori et al. [26]) have been used to study the effects of the loading rate and confining pressure on the mechanical properties of conditioned soils, and specially designed rheometers have been used to test the characteristic parameters (such as yield stress and viscosity) (Karmakar [27]; Meng et al. [28]) of muck. In general, the results of this type of test method can simulate the influence of the pressure environment of the working chamber on the strength of muck to some extent and can characterize the viscoplastic parameters of muck.

The above results have advanced the understanding of the mechanical properties of conditioned soil. To more accurately simulate the mechanical environment and stress state of the muck in the screw conveyor of the EPB shield, this paper developed a shield screw conveyor model. By analyzing the forces and motion of the soil microbody according to the steady-state equilibrium conditions of moment of momentum, the equivalent spiral shear stress calculation model of the EPB shield screw conveyor is derived. The test results confirm the validity and rationality of the model.

## Calculation model for the equivalent helical shear stress of conditioned soil

For the convenience of analysis, the symbols used in the paper are listed in S1 Table.

### Description of the spiral structure

**Fig 2:**
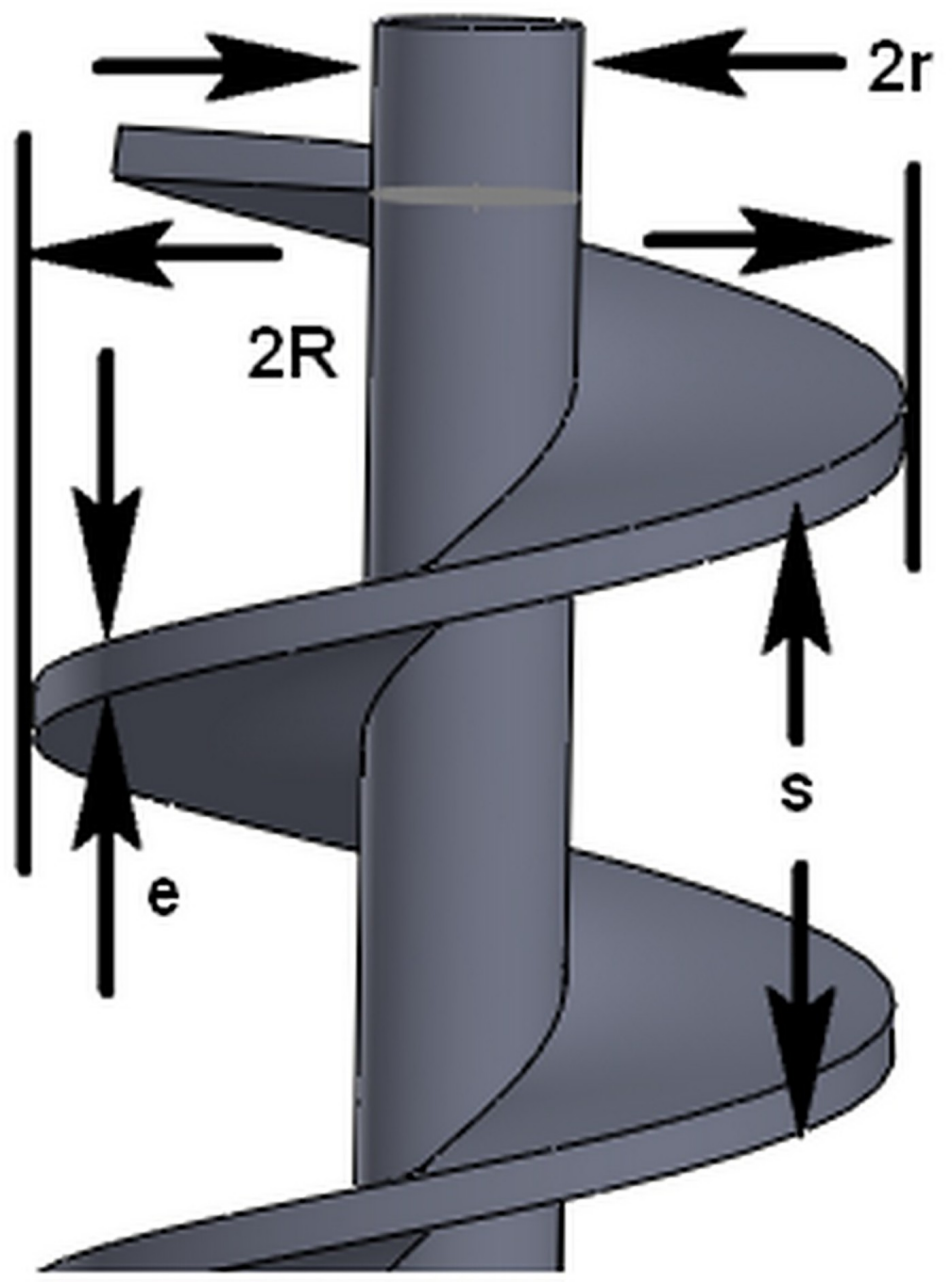
Schematic view of the screw conveyor structure.

The parameters of the spiral blade and screw shaft of an EPB shield screw conveyor are shown in Fig 2.

**Fig 3:**
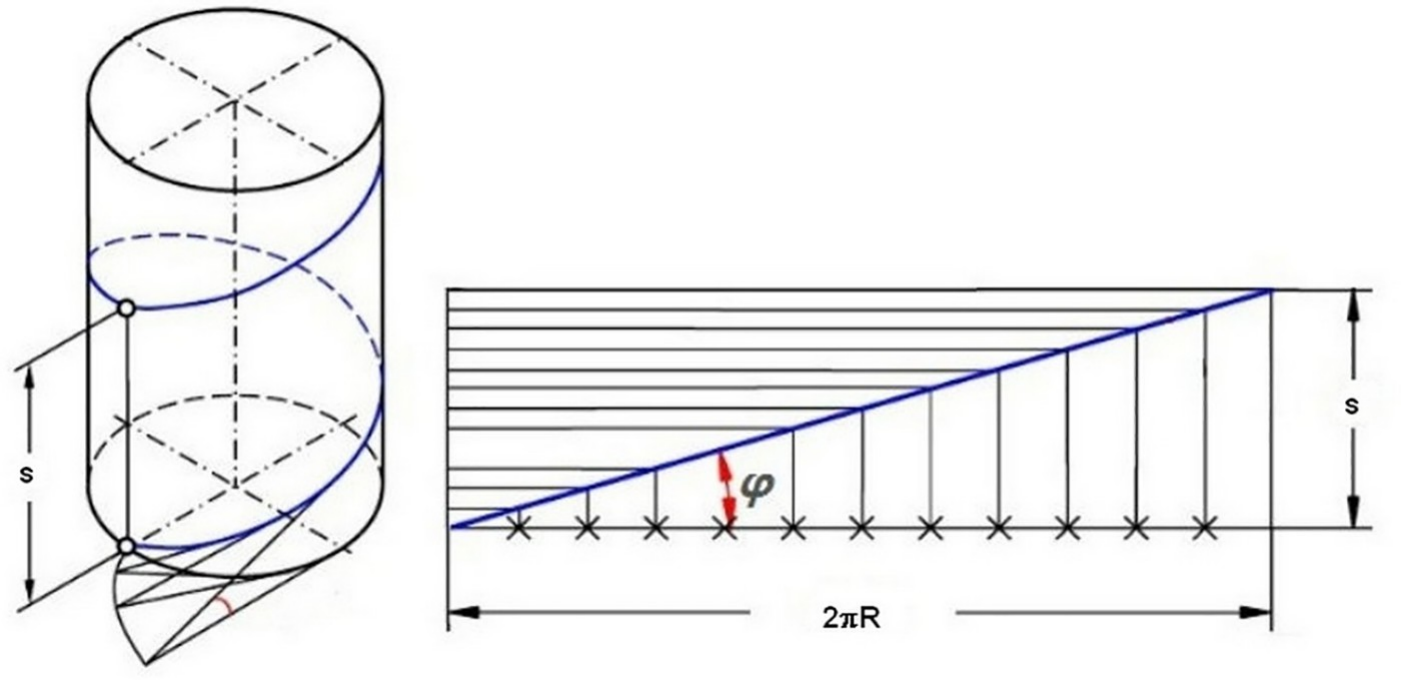
The definition of the helical angle.

The angle between the tangent of any point on the spiral blade and its horizontal projection is the helix angle. Unfolding the spiral of a pitch, the spiral is the oblique side of the right-angled triangle, the pitch is the cathetus of the right triangle, and the spiral is another cathetuswhen it is projected in the horizontal direction. The unfolding result is shown in Fig 3. From this figure, the helix angle *φ* of any point on the spiral blade can be derived.

$$\sin\varphi =\frac{dy}{dl}$$ (1)

The spiral angle of the outer edge of the spiral blade is defined by Eq (2), and the definition of the helix angle at the spiral axis is given by Eq (3).

$$\varphi =\arctan\frac{s}{2\cdot \pi \cdot R}$$ (2)

$$\varphi_{s}=\arctan\frac{s}{2\cdot \pi \cdot r}$$ (3)

### Stress and motion analysis of the soil microbody

**Fig 4:**
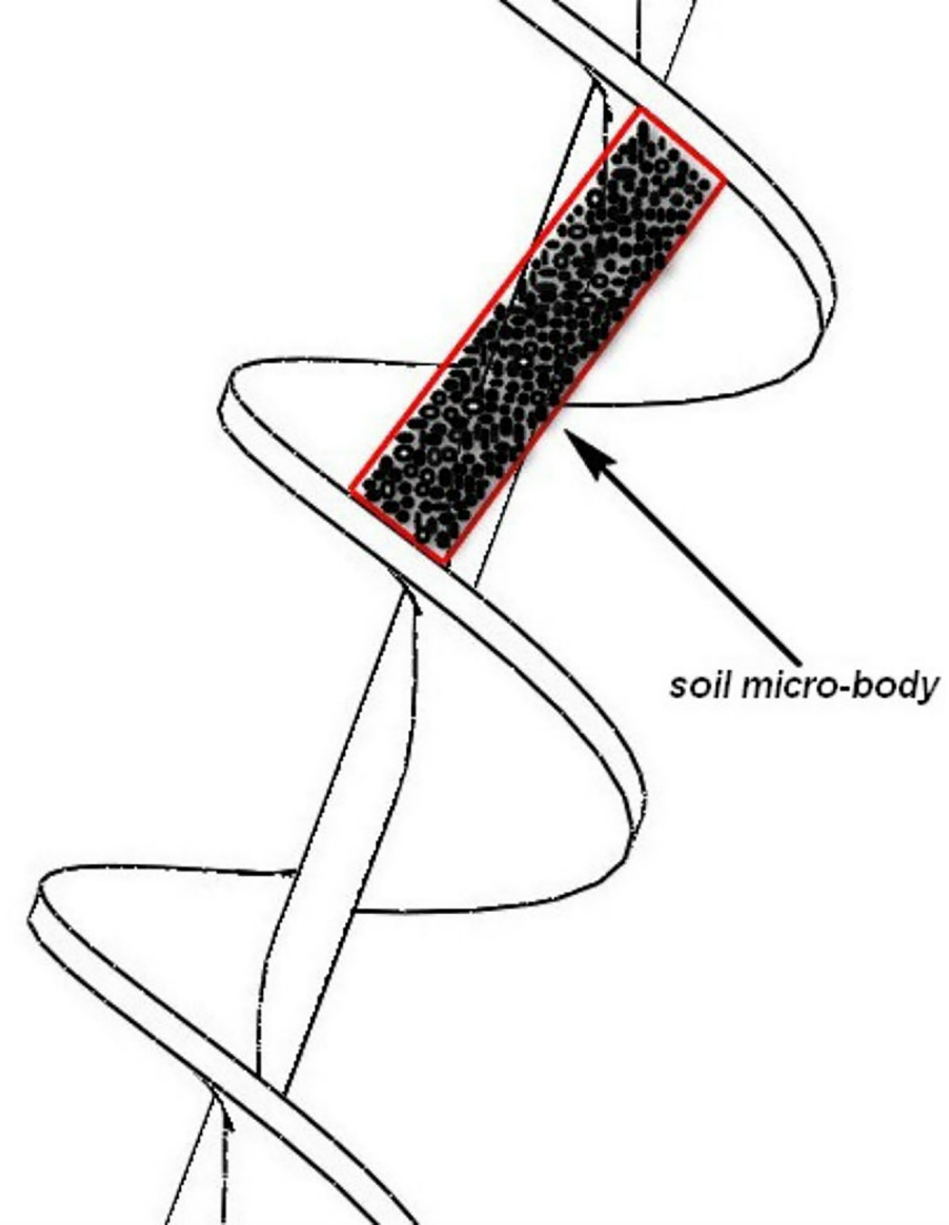
The studied object.

**Fig 5:**
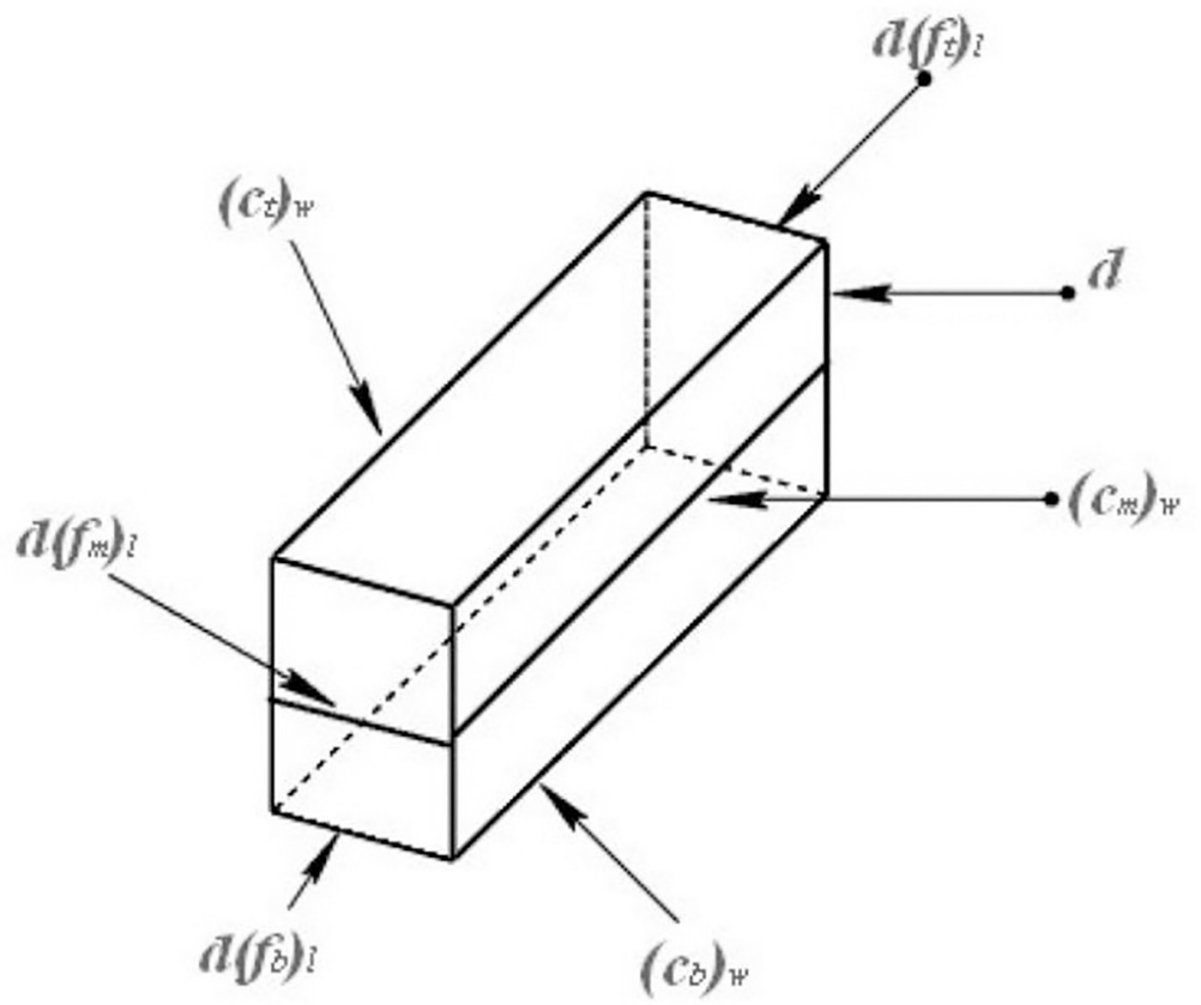
The size of the soil microbody.

It is assumed that the conditioned sandy soil is a homogeneous fluid body and is isotropic. As shown in Fig 4, the soil microbody in the spiral groove is taken as the research object. The size of the microbody is defined in Fig 5.

Eqs (4) to (10) are derived from the relationships among the sizes of the microbody.

$$\sin\varphi ={\frac{dy}{d\left(f_{t}\right)}}_{l}$$ (4)

$$\sin\varphi_{m}={\frac{dy}{d\left(f_{m}\right)}}_{l}$$ (5)

d(ft)ld(fm)l=RR−H2 (6)

d(fb)ld(fm)l=rR−H2 (7)

$${\left(c_{t}\right)}_{w}=(s-e)\cdot \cos\varphi$$ (8)

$${\left(c_{m}\right)}_{w}=(s-e)\cdot \cos\varphi_{m}$$ (9)

$${\left(c_{b}\right)}_{w}=(s-e)\cdot \cos\varphi_{s}$$ (10)

**Fig 6:**
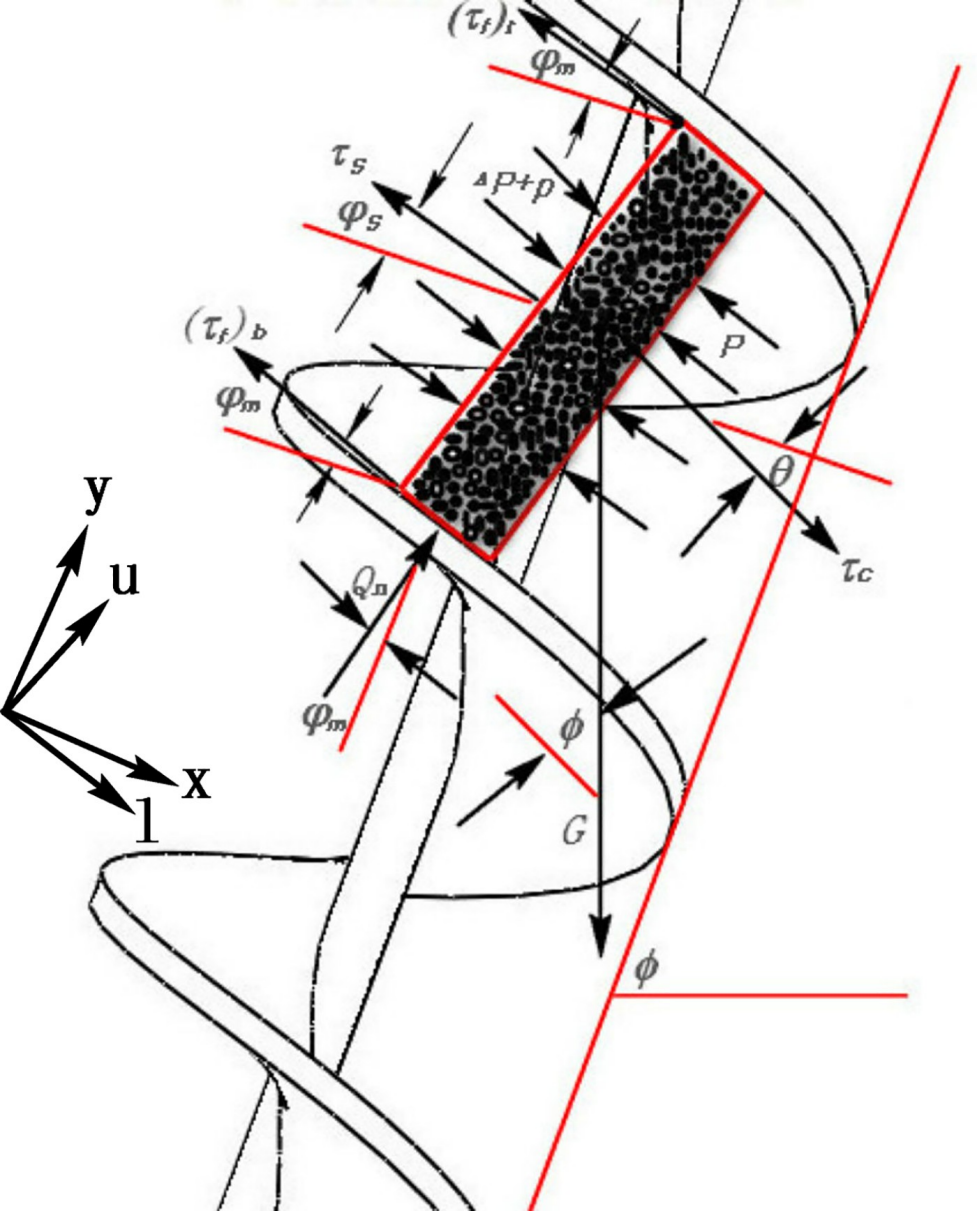
The forces acted on the soil microbody.

During the conveying of the muck by the shield screw conveyor, the soil in the spiral groove is subjected to seven forces: resultants of shear stresses (shear stress of the upper spiral blade (*τ*_*f*_)_*t*_, shear stress of the lower spiral blade (*τ*_*f*_)_*b*_, shear stress of the helical axis *τ*_*s*_, and shear stress of the spiral shell *τ*_*c*_), the combined pressing force *Q*_*n*_ of the upper and lower spiral blades, the pressure *P* in the direction of the spiral groove and the weight *G* of the soil microbody. Fig 6 shows the forces acted on the soil microbody. The two-axis system is defined in Fig 6; the (*x*, *y*) axis is perpendicular and parallel to the screw shaft, and the (*l*,*u*) axis is parallel and perpendicular to the screw blades.

**Fig 7:**
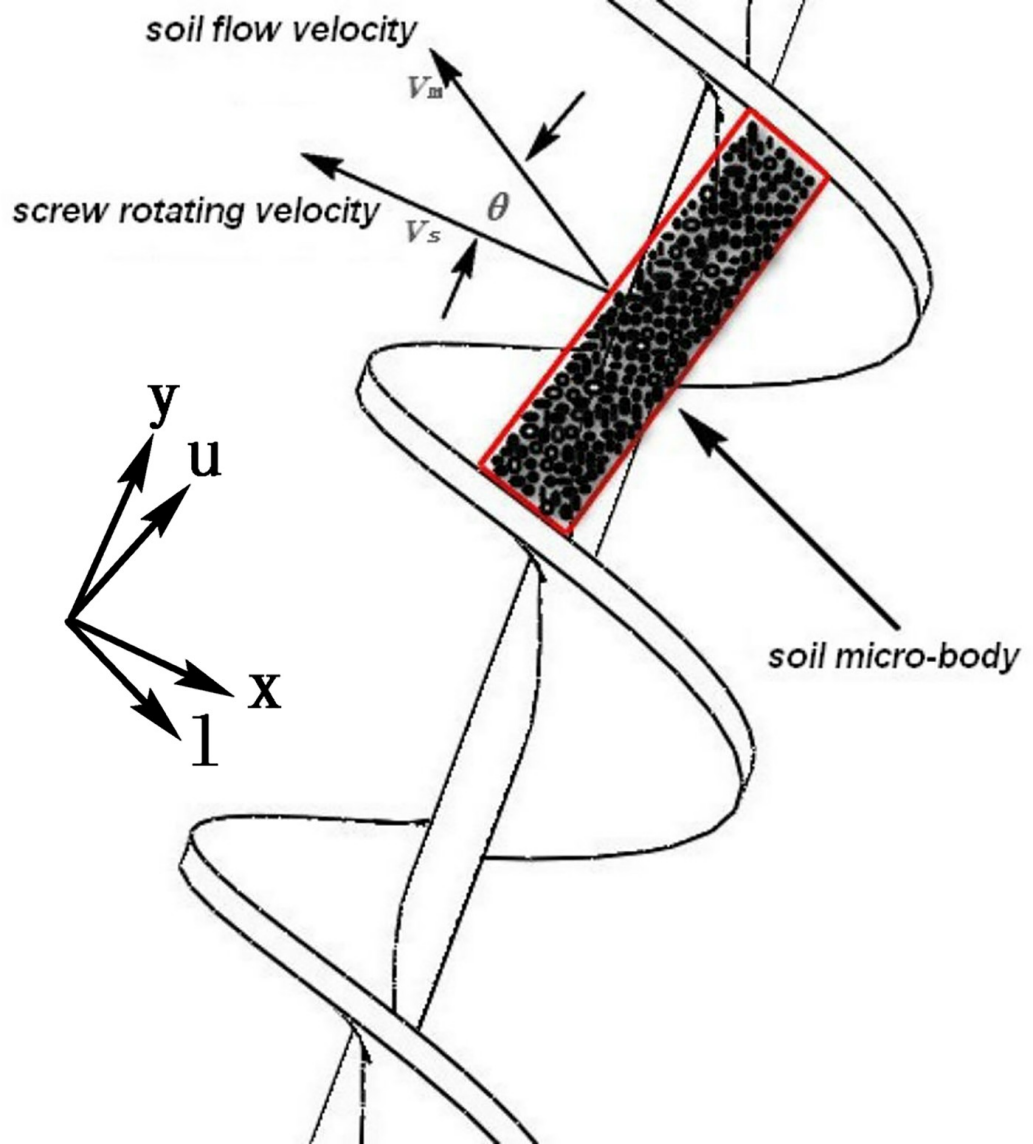
The movement of the soil microbody.

During the normal tunneling process of the shield, due to the relative movement between muck and both the spiral groove and the spiral shell, friction shear stress is generated between the soil in the spiral groove and the spiral casing, as well as the spiral shaft and the spiral blade. Under the action of the helical axis and the shear stress of the spiral blade, the soil microbody has a linear velocity *v*_*m*_ following the rotation of the spiral blade *v*_*s*_, and the angle between the velocity *v*_*m*_ and the direction of the vertical spiral axis is *θ*, as shown in Fig 7.

**Fig 8:**
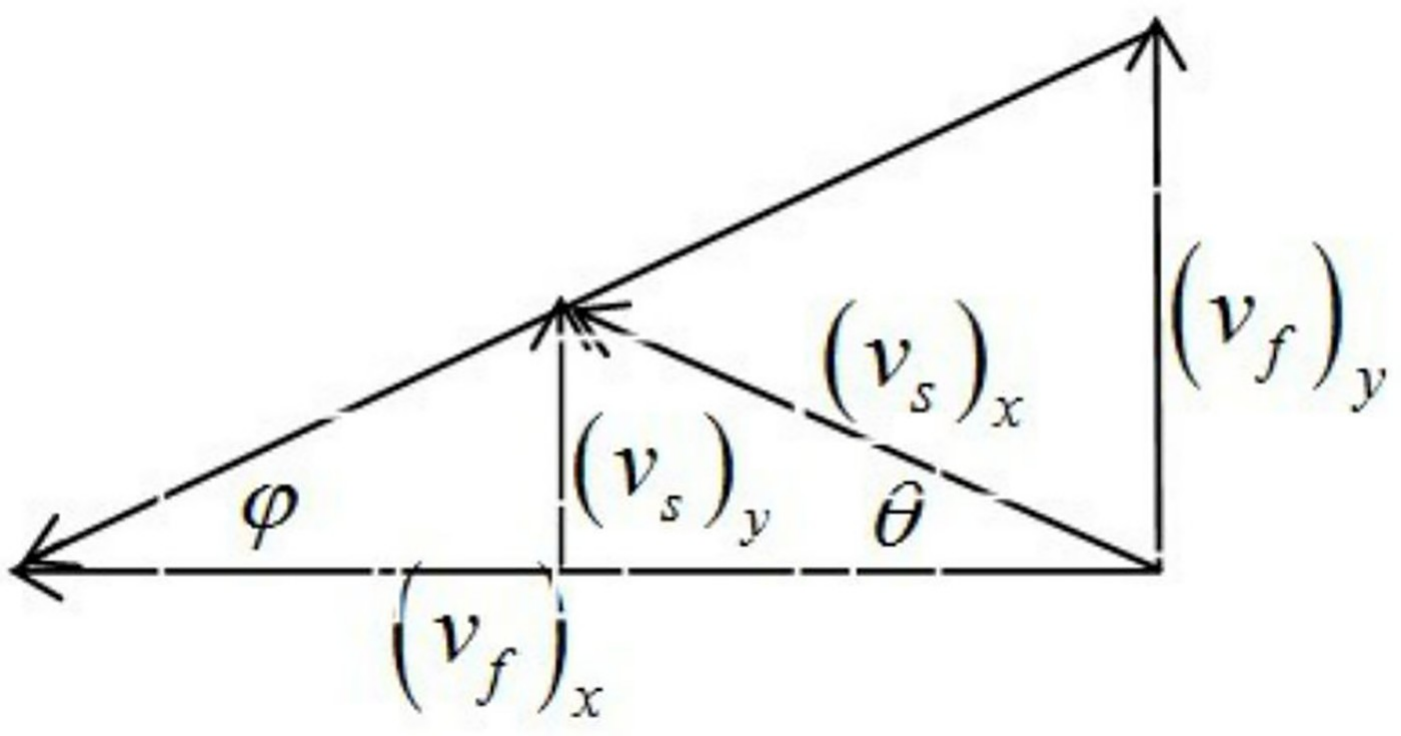
The velocity vector between the soil microbody and the blade.

Suppose that the rotational speed component of the blade tangent to the spiral shell is (*v*_*f*_)_*x*_ and (*v*_*f*_)_*y*_ is the axial velocity component of the blade. The relative velocity of the soil microbody and the spiral casing is (*v*_*s*_)_*x*_, *θ* is the angle between the velocity and the direction of the vertical spiral axis, and (*v*_*s*_)_*y*_ is the axial velocity component of the soil microbody. The velocity vector relationship between the spiral blade and the soil microbody is shown in Fig 8.

The rotational speed component of the spiral blade tangent to the spiral casing is

(vf)x=(n⋅2π⋅R60) (11)

The speed of the spiral blade along the axis is

$${\left(v_{f}\right)}_{y}={\left(v_{f}\right)}_{x}\cdot \tan\varphi$$ (12)

Eqs (13) and (14) can be derived from Fig 8.

$$\left|\frac{{\left(v_{s}\right)}_{y}}{\tan\theta }\right|+\left|\frac{{\left(v_{s}\right)}_{y}}{\tan\varphi }\right|=\left|{\left(v_{f}\right)}_{x}\right|$$ (13)

$$\left|{\left(v_{s}\right)}_{y}\right|=\left|{\left(v_{f}\right)}_{x}\right|\cdot \frac{\tan\theta \cdot \tan\varphi }{\tan\theta +\tan\varphi }$$ (14)

Using Eqs (12) and (14), the conveying efficiency η of the screw conveyor is

$$\eta =\frac{{\left(v_{s}\right)}_{y}}{{\left(v_{f}\right)}_{y}}=\frac{\tan\theta }{\tan\theta +\tan\varphi }$$ (15)

Referring to the geometry of the screw conveyor, the maximum volume delivery in theory is

$$Q_{M}=\frac{n}{60}\cdot s\cdot \pi \cdot \left(R^{2}-r^{2}\right)$$ (16)

The actual delivery volume is

$$Q=\eta \cdot Q_{M}$$ (17)

The following equations can be derived from Eqs (15) and (17)

$$\frac{Q_{M}}{Q}-1=\frac{\tan\varphi }{\tan\theta }$$ (18)

As a result, the calculation formula of the flow direction of the muck can be obtained as follows:

$$\theta =\arctan\left[\tan\varphi \cdot {\left(\frac{Q_{M}}{Q}-1\right)}^{-1}\right]$$ (19)

## Derivation of the equivalent spiral shear stress formula

When the shield machine is smoothly boring through soil, the screw conveyor quantitatively discharges the conditioned soil. As the muck is spirally moved upward in the spiral groove, a reverse helical drive torque is generated under the action of the force in the direction of the opposite spiral rotation. The equivalent spiral shear stress calculation model can be derived from the equilibrium of moment of momentum in the direction of the vertical spiral axis.

It can be seen from the motion and the forces of the research object (Figs 5–7) that only the friction force between the muck and the casing and the weight of the soil have the components in the opposite direction of the rotation. According to the momentum equilibrium condition of the research object along the vertical spiral axis direction, the following formula can be written:

$$dT=-\tau_{c}\cdot {\left(c_{t}\right)}_{w}\cdot d{\left(f_{t}\right)}_{l}\cdot \cos\theta \cdot R-\rho \cdot g\cdot d\cdot {\left(c_{m}\right)}_{w}\cdot d{\left(f_{m}\right)}_{l}\cdot \cos\phi \cdot \left(R-\frac{d}{2}\right)$$ (20)

The negative sign on the right side of Eq (20) indicates that the weight of the muck’s microbody and the shear stress of the spiral shell perpendicular to the direction of the spiral axis are opposite to the direction of the rotational torque.

Substituting Eqs (4), (5), (8) and (9) into Eq (20), the following equation can be obtained (to simplify the analysis, the thickness *e* of the spiral blade is ignored):

$$dT=-\tau_{c}\cdot \frac{s}{\tan\varphi }\cdot R\cdot \cos\theta \cdot dy-\rho \cdot g\cdot d\cdot \frac{s}{\tan\varphi_{m}}\cdot \left(R-\frac{d}{2}\right)\cdot \cos\phi \cdot dy$$ (21)

Ignoring the difference between the helix angle *φ* of the outer edge of the spiral blade and the average helix angle *φ*_*m*_ of the spiral blade and using Eq (2), Eq (21) can be further simplified to

$$dT=-2\pi \cdot \tau_{c}\cdot R^{2}\cdot \cos\theta \cdot dy-2\pi \cdot \rho \cdot g\cdot d\cdot {\left(R‑\frac{d}{2}\right)}^{2}\cdot \cos\phi \cdot dy$$ (22)

Eq (22) is integrated along the direction of the helix axis, the length of which is *L*. Assuming the muck filling rate is to be 100% (this is in line with reality), the equivalent spiral shear stress formula is available

$$\tau_{c}=-\frac{T}{2\pi \cdot R^{2}\cdot \cos\theta \cdot L}-\frac{\rho \cdot g\cdot d\cdot {\left(R-\frac{d}{2}\right)}^{2}\cdot \cos\phi }{R^{2}\cdot \cos\theta }$$ (23)

According to Eq (23), the calculated shear stress is affected by the helical geometric parameters, the density of the conditioned muck, the angle of the screw installation, and the parameters of the operating state. Through this formula, the shear stress at the interface between the conditioned soil and the conveyor casing can be calculated for given torques and flow directions of the muck. When *θ* is 0°, the screw conveyor is clogged, and the muck does not move in the direction of the screw axis. The shear stress when the muck is clogged can be calculated from Eq (23); thus, the quantitative analysis and suggested calculation parameters can be provided for preventing the blockage of the shield screw conveyor. Under the premise of determining the geometry and installation inclination angle of the shield screw conveyor machine, the shear stress of the conditioned soil is proportional to the screw machine driving torque and the flow angle of the muck. In other words, the more rigid the muck is, the greater the required driving torque and the higher the shear stress at the same soil flow angle.

It is seen that the muck density is of great importance for the stress created at the interface between the conveyor casing and the conditioned soil. Then how to get the desired density is to be concerned. From the difference of the density between the soil and the conditioned soil (the muck), the conditioned muck relates with the specialized additives and the injection amount of the specialized additives, both of which can be decided quantitatively in the laboratory.

## Indoor model machine test

To test the validity of the model for calculating the equivalent shear stress of the conditioned soil, a series of muck conveyance tests were conducted on the shield screw conveyor model machine, and the difference between the measured values of the model test and the theoretical calculation results was compared and analyzed. The influence of different factors on the shear stress of conditioned soil was discussed.

### Shield screw conveyor model machine

**Fig 9:**
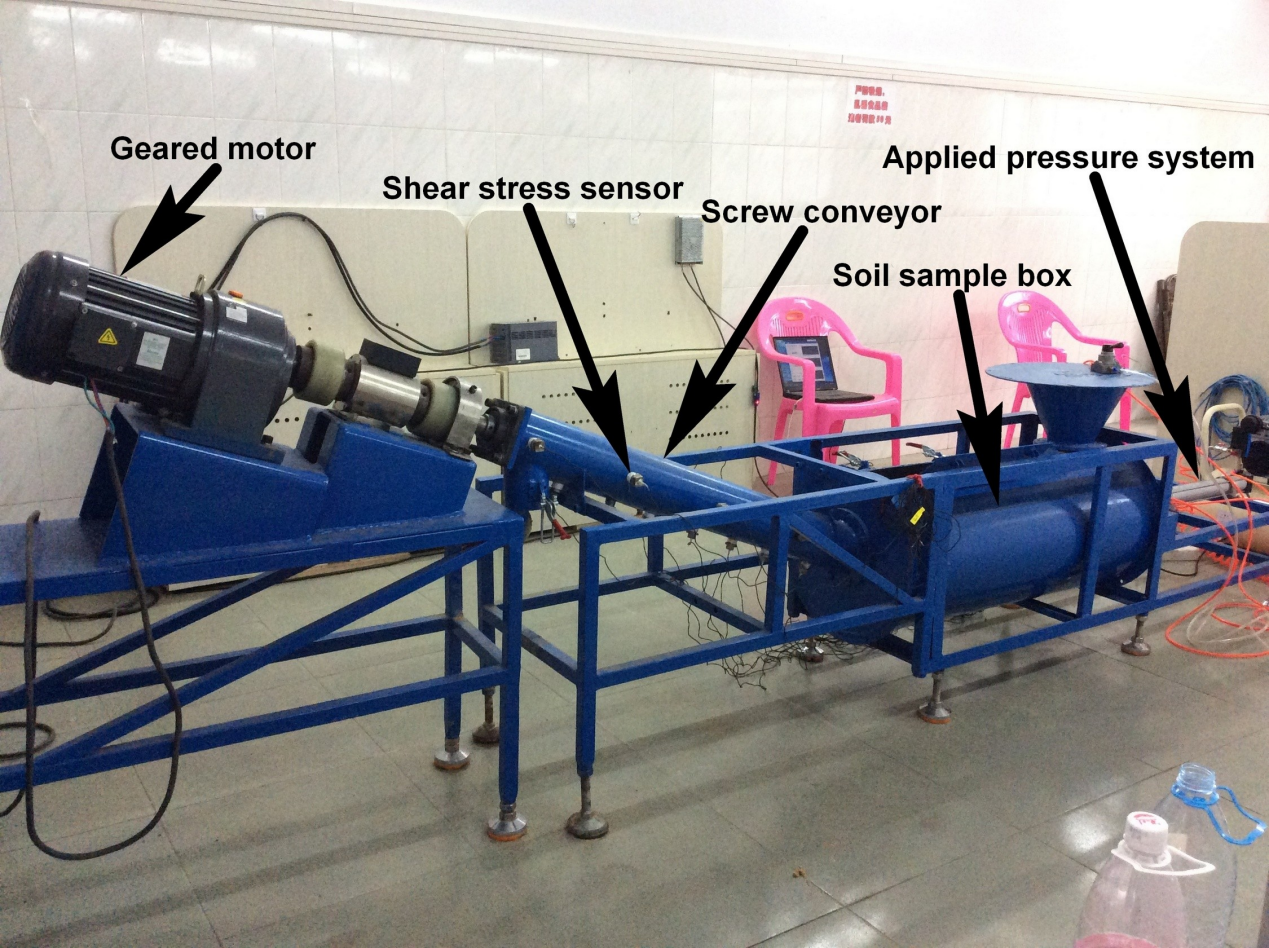
Screw conveyor model machine.

The indoor model test device is shown in Fig 9. The model machine system consists of a soil sample box, a tilted spiral tube with a screw inside, a coupling, a torque sensor, a geared motor, a frequency converter and a horizontally applied pressure system in the soil sample box. Load cells, stress sensors and pore sensors are installed outside the spiral tube for measuring the normal stress, the pore water pressure, and the shear stress in the direction of the parallel and vertical spiral axes. Motor drive torque and speed are collected by the torque sensor. According to the soil transportation situation in the soil box, the muck transportation rate is calculated. The specific technical parameters of each component are shown in Table 1. The conveyor model was modeled after the screw conveyors installed in the shield machines widely used in Beijing tunnel construction. For convenience of easy operation and manufacture, the minification ratio of 1:8 was adopted, resulting in the parameters listed in Table 1.

**Table 1:** Parameters of the model screw conveyor.

Groove depth (mm)	Spiral blade diameter 2*R*(mm)	Spiral shaft diameter 2*r*(mm)	Pitch of the screw *S*(mm)	Screw installation angle (°)	Screw length (mm)	Inner diameter of casing (mm)	Blade thickness (mm)	Rotation speed (rpm)	Volumetric delivery rate Q_M_ (m^3^/h)	Testing pressure *P*(MPa)
29.5	102	43	102	17.66	1000	108	3	6	0.25	0.3

### Experiment material

Standard sand was selected for the test, which was manufactured by Xiamen Iso Standard Sand Co., Ltd. After screening with a particle size of 2 mm, the particle sizes were between 0.075 and 2.0 mm, the silica (SiO_2_) content was greater than 96%, and the mud content (including soluble salts) was not more than 0.2%. The physical and mechanical indicators are shown in Table 2, which are provided by the supplier. A series of slump tests were conducted using this standard sand to determine the workability of the muck and to determine reasonable additives and ratios.

**Table 2:** Geotechnical indices of standard sand.

Specific gravity of sand G_s_	Average grain size D_50_ (mm)	Uniformity coefficient C_u_	Curvature coefficient C_c_	Maximum void ratio e_max_	Minimum void ratio e_min_	Internal friction angle φ (°)
2.62	0.82	1.8	0.82	0.72	0.4	33

**Fig 10:**
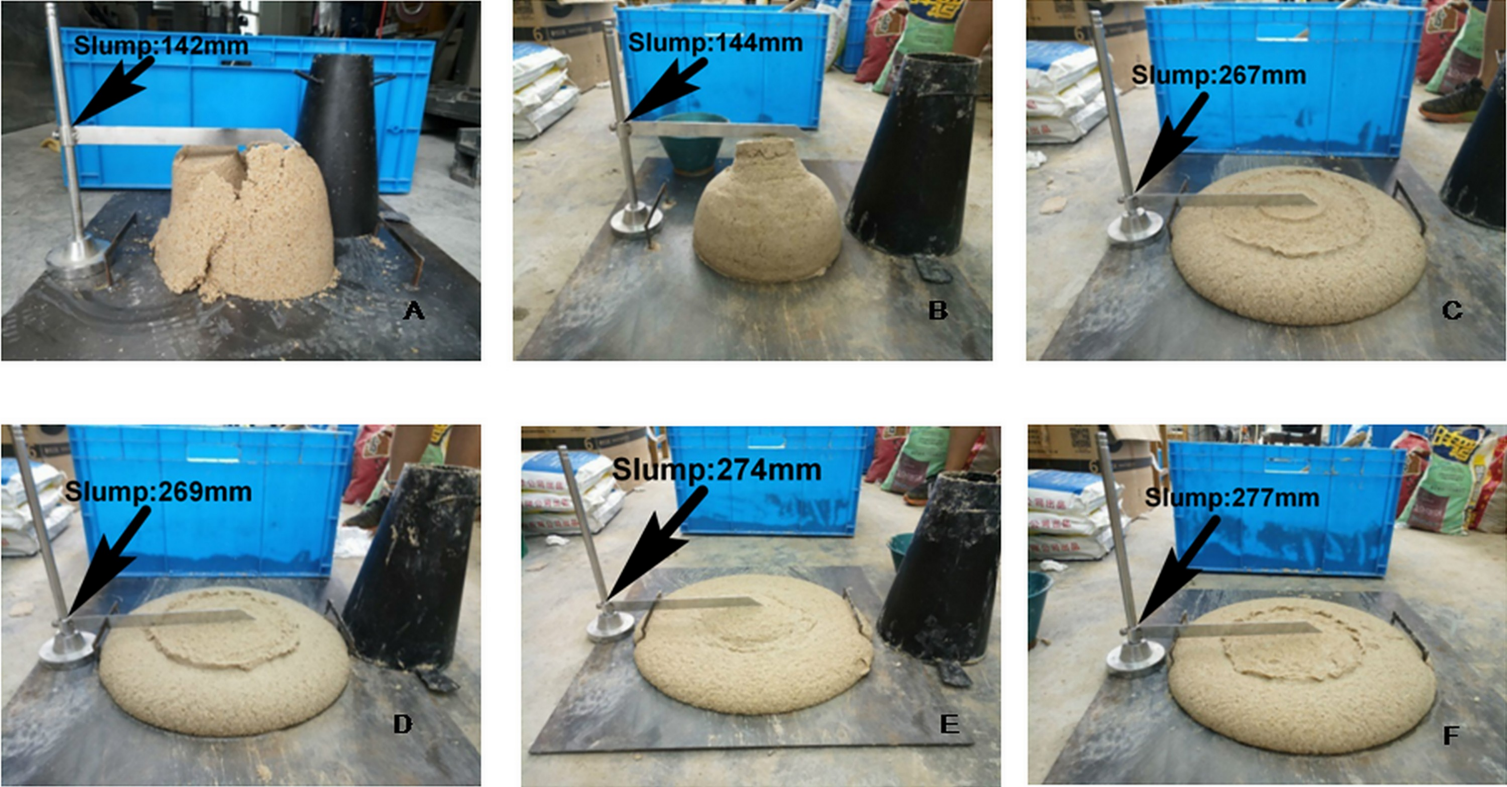
The slump tests of composite additive conditioning soil. (A) Only 15% bentonite slurry. (B) 15% bentonite slurry with 10% foam solution. (C) 15% bentonite slurry with 20% foam solution. (D) 15% bentonite slurry with 30% foam solution. (E) 15% bentonite slurry with 40% foam solution. (F) 15% bentonite slurry with 50% foam solution.

The foaming agent solution can enhance the fluidity of the muck, and the high-quality bentonite slurry has a good cohesive force. When too much slurry is added, the fluidity of the sand will also increase. According to this feature, a sodium-based bentonite slurry with a mass ratio of 1:5 is selected, with a slurry volume injection ratio of 15%. At this time, the sand has a certain adhesive force, but it is still loose and brittle, and the fluidity is poor. Subsequently, different amounts of foam with a concentration of 7% are added, increasing the blending ratio by 10% each time until the desired fluidity effect is achieved (the optimal slump of sandy soil is in the range of 100-200mm [29]).The test results of standard sand conditioned by bentonite slurry and foam with different injection amount are shown in Fig 10.

**Fig 11:**
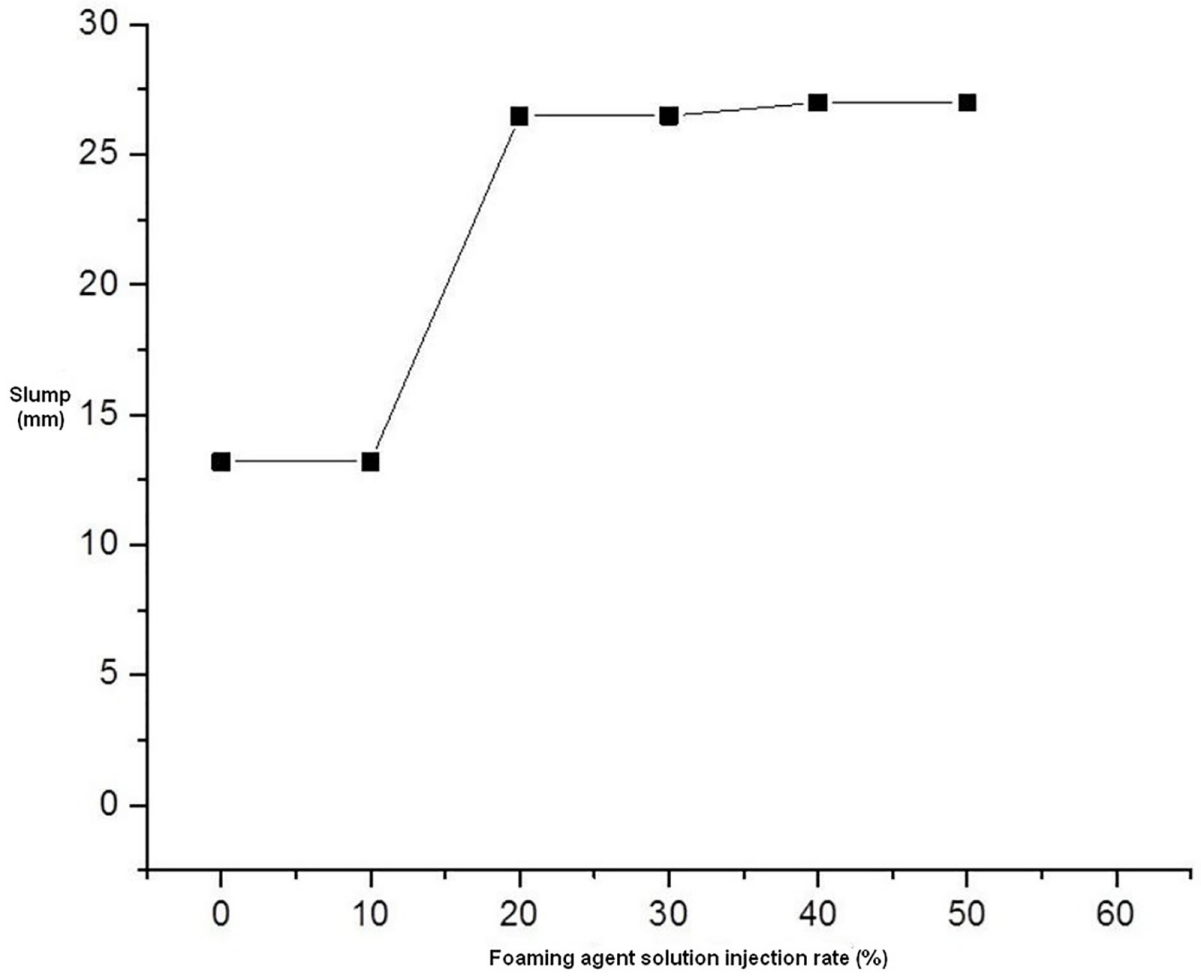
Slumps under different additive injection ratios.

It can be seen from the test results in Fig 10 that, when only 15% bentonite slurry is added, the sand is in a relatively loose state. The soil sample can reach a relatively good flow state after adding 10% foam. The variation of the conditioned sandy soil slump with different foam solution injection ratios is shown in Fig 11.

Based on the above test results, the soil conditioning parameters used in the model machine test are a concentration of 7% foaming agent solution with a blending ratio of 10% and a sodium-based bentonite slurry with a water-soil quality of 5:1 and a blending ratio of 15%. The conditioned muck density was 1452 kg/m^3^.

### Test results

The helical shear stress of each measuring point is synthesized by the horizontal component and the vertical component, and is calculated according to Eq (24).

$$\tau_{r}=\sqrt{{\left(\tau_{y}\right)}^{2}+{\left(\tau_{x}\right)}^{2}}$$ (24)

**Fig 12:**
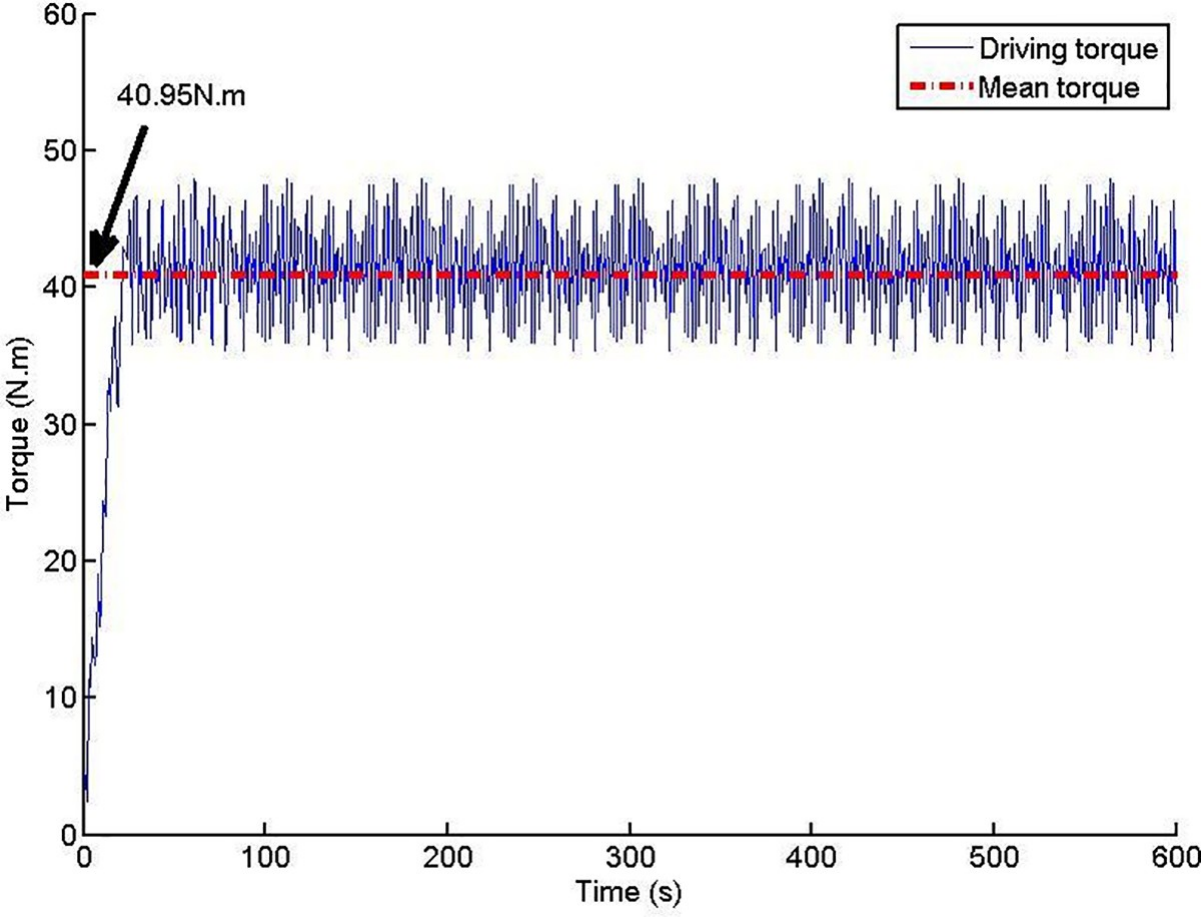
Driving torque schedule.

The laboratory test was repeated three times under the same test conditions. The data measured below are the average of the three measurements. After the working state of the shield screw conveyor model machine is stable, the volume transportation rate of the conditioned soil becomes constant. According to the volume of the soil sample in the sand sample box and the volume of the muck discharged from the outlet, the average transporting rate of the muck is calculated to be 0.18 (m^3^/h); thus, the average transport efficiency of the model machine is 79%. The driving torque is shown in Fig 12. After the model machine runs for approximately 30 seconds, the driving torque enters the steady-state range. The average torque is 40.95 *N* ⋅ *m*. According to Eq (19), the flow angle of the muck is calculated to be 51°. From Eq (23), the helical shear stress at the interface between the modified soil and the conveyor casing can be calculated to be 4.15 *KPa*.

**Fig 13:**
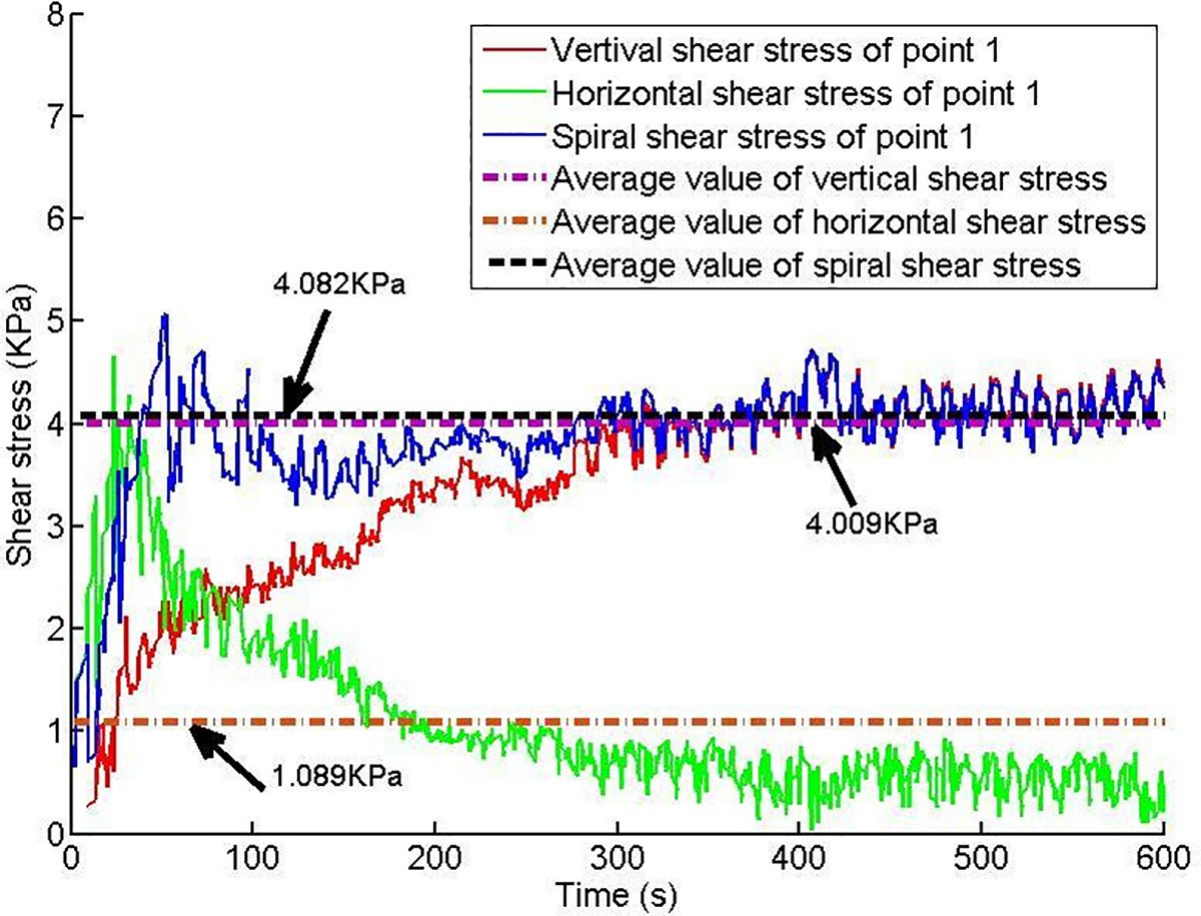
Shear stress of testing point 1.

**Fig 14:**
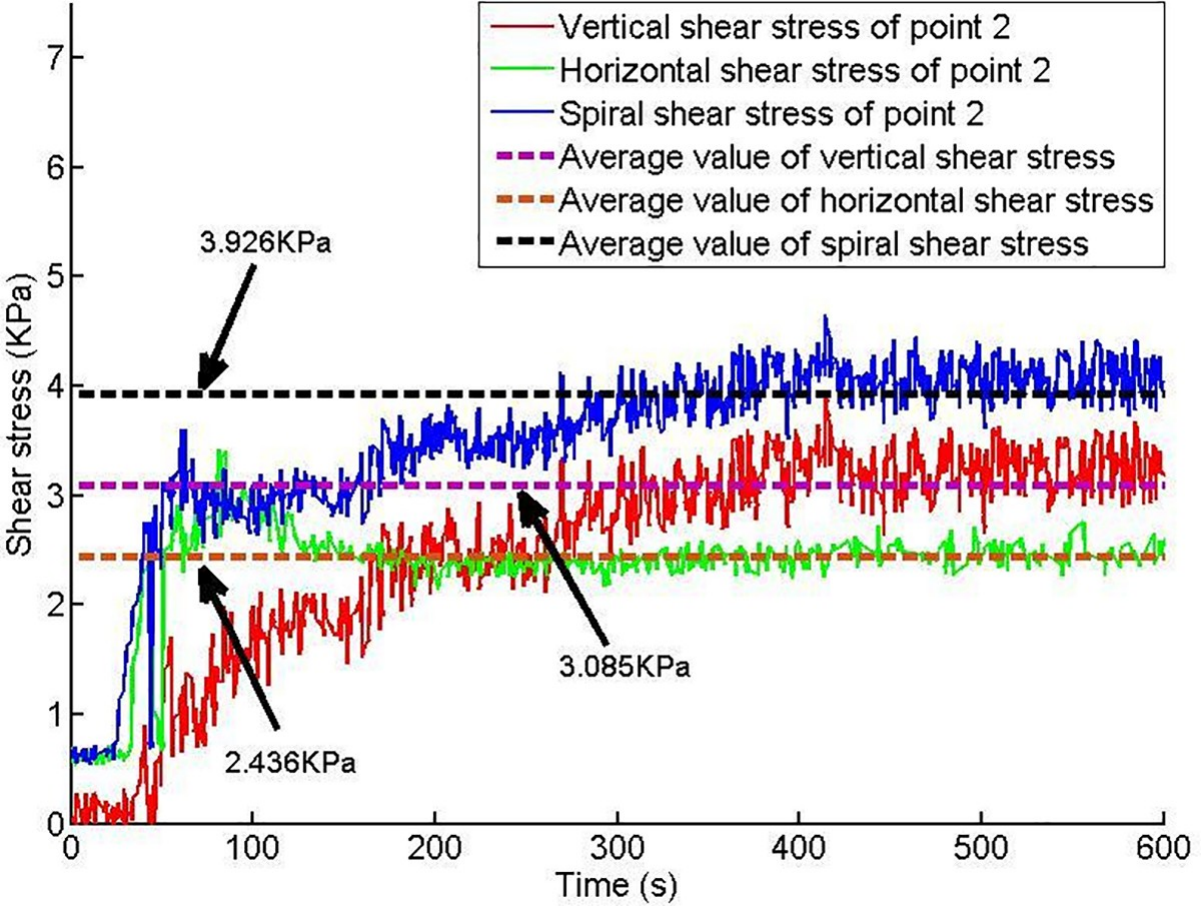
Shear stress of testing point 2.

**Fig 15:**
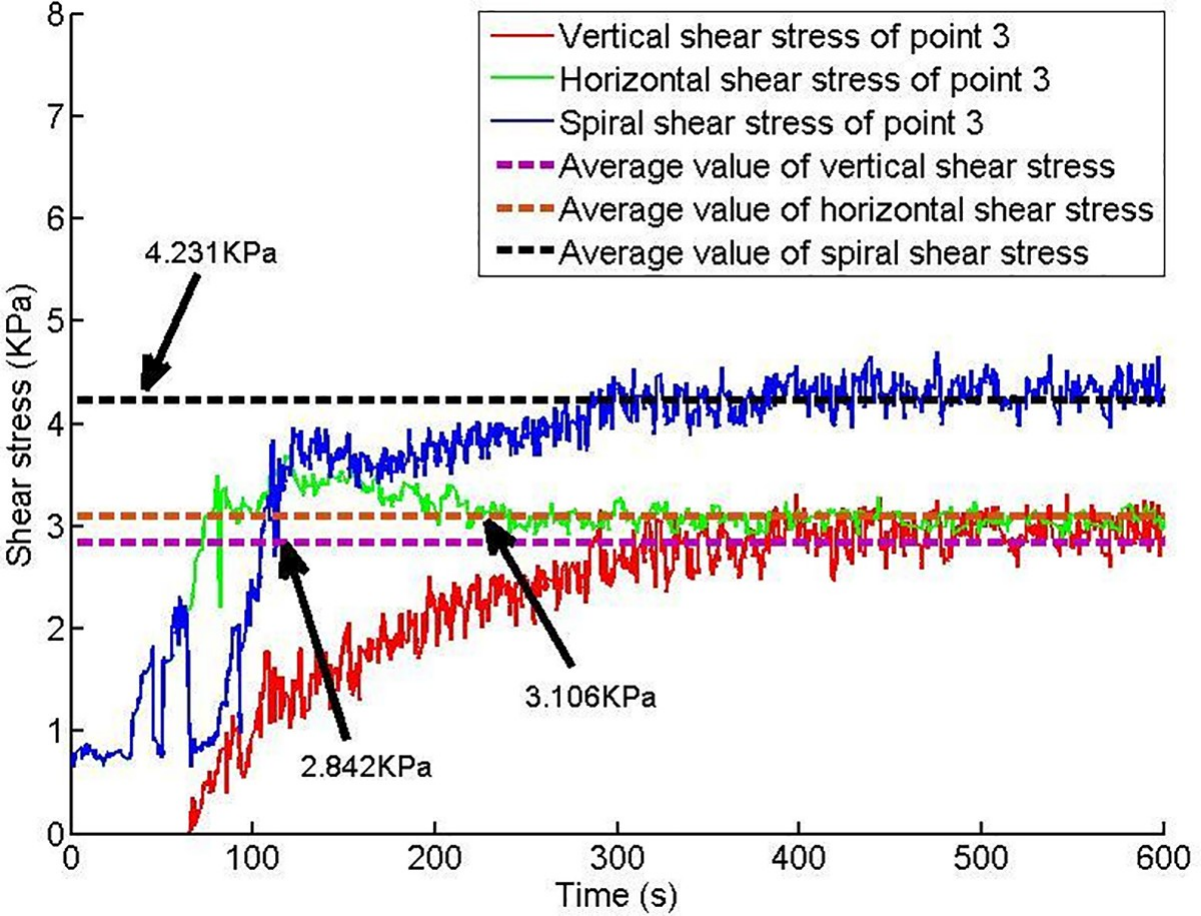
Shear stress of testing point 3.

**Fig 16:**
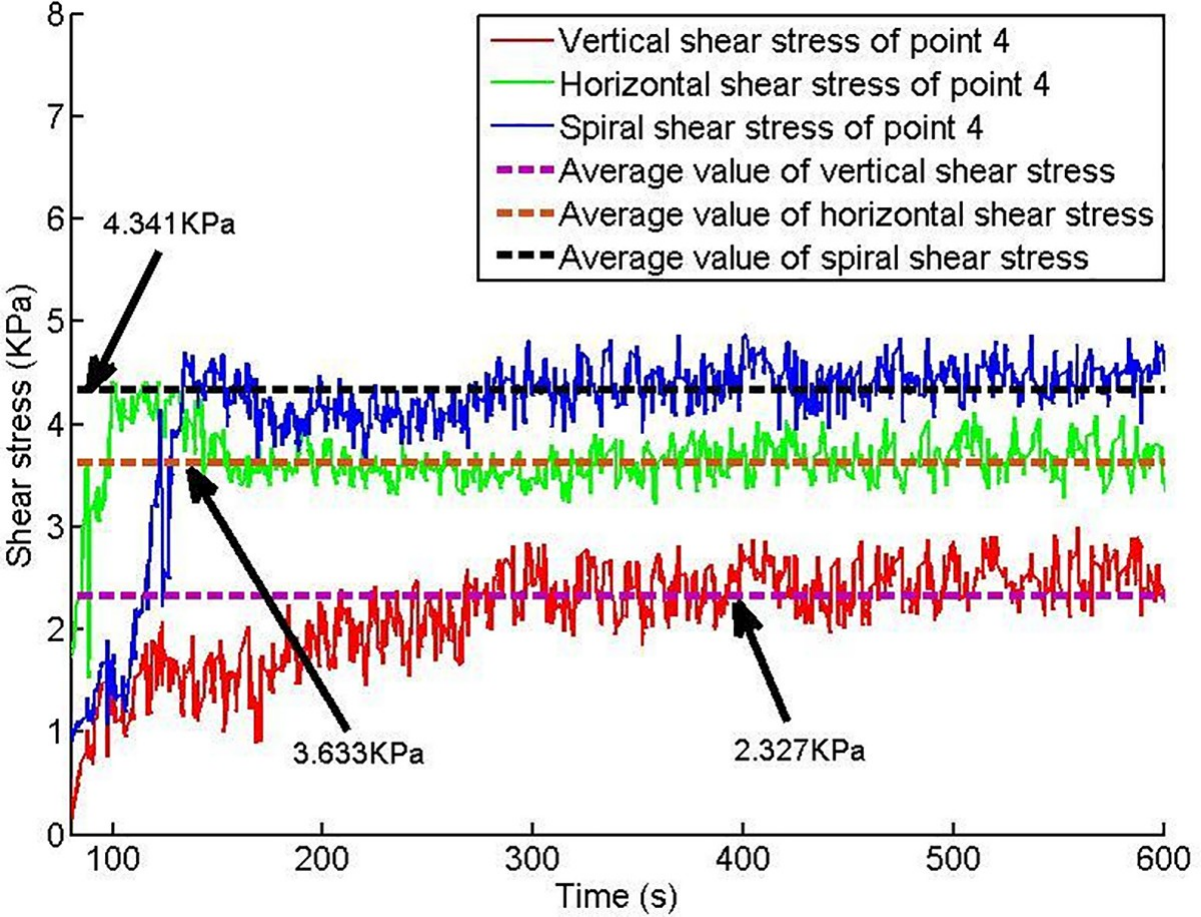
Shear stress of testing point 4.

**Fig 17:**
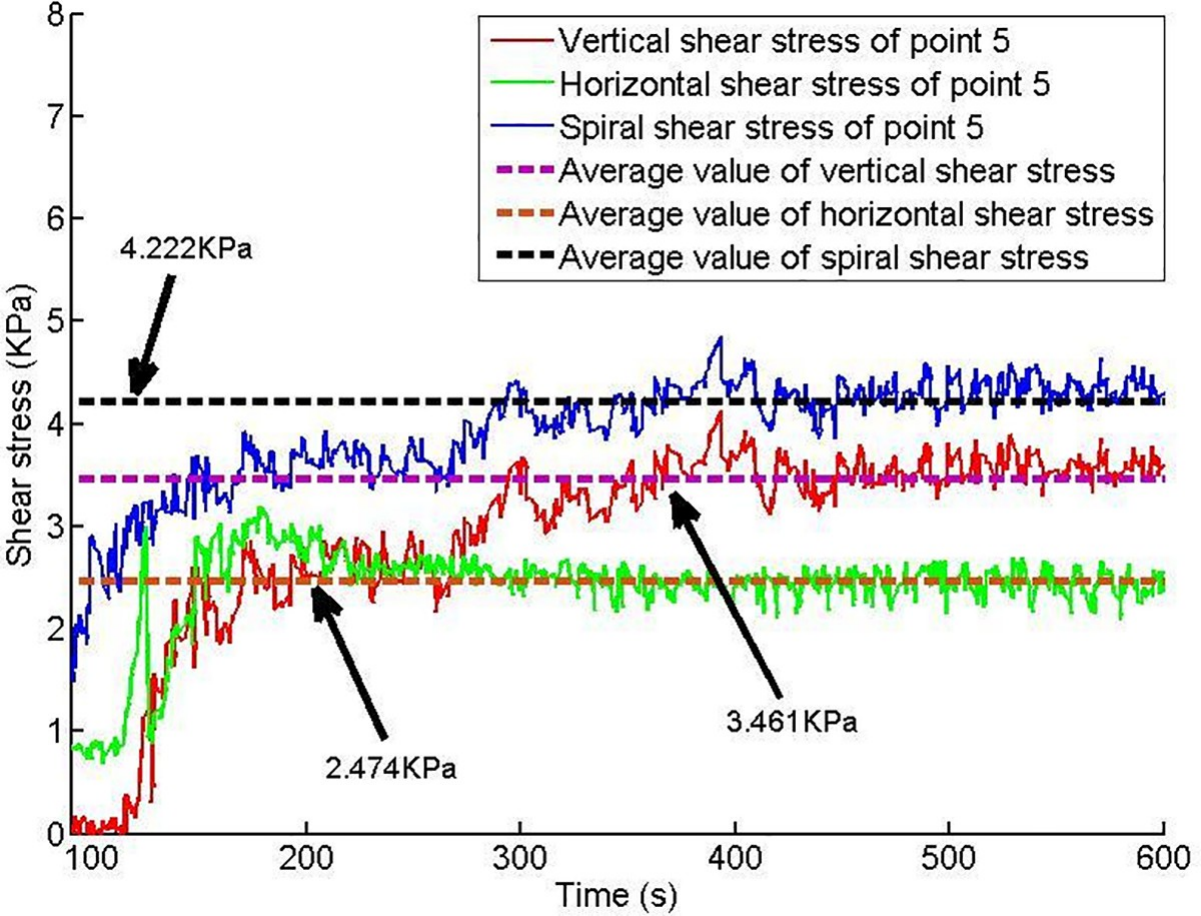
Shear stress of testing point 5.

**Fig 18:**
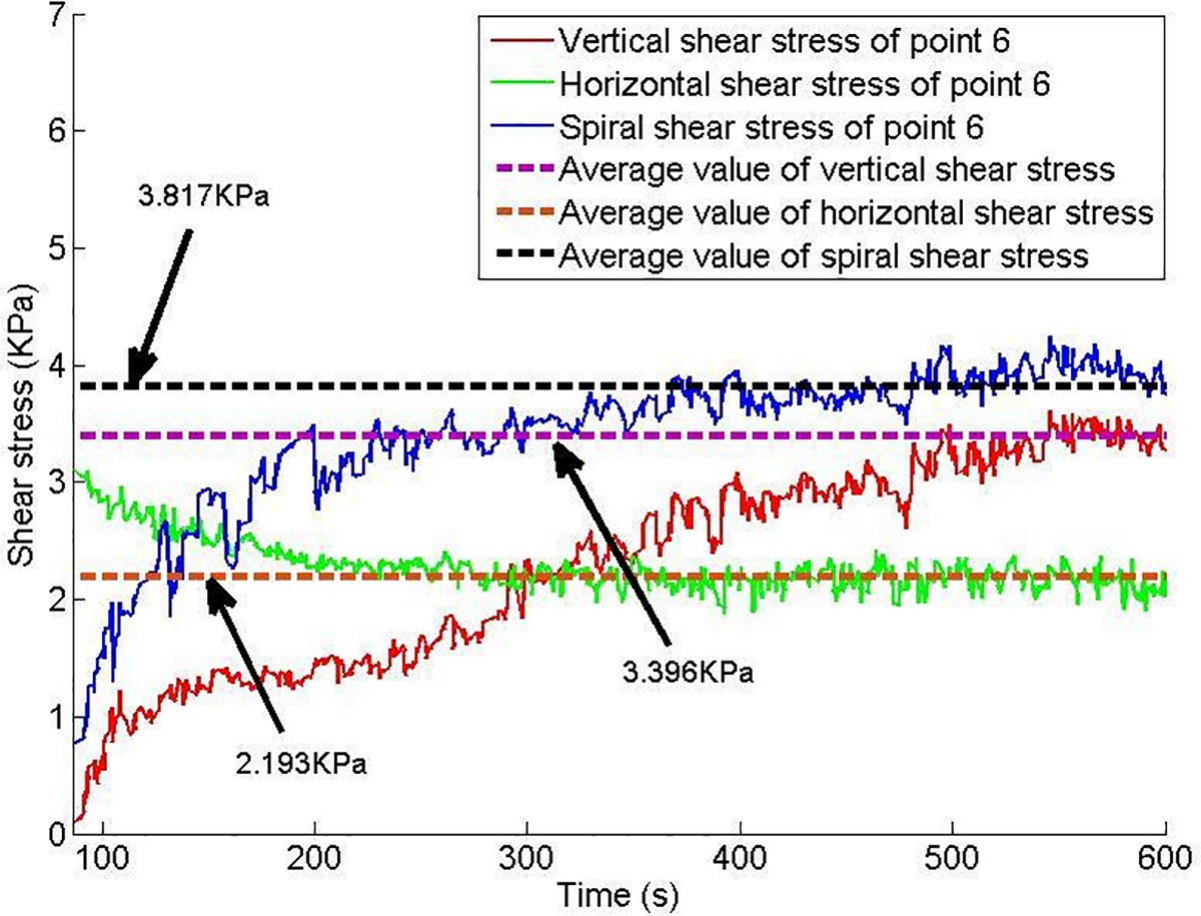
Shear stress of testing point 6.

Twelve shear stress measuring points are arranged on the conveyor casing, and the shear stress of each measuring point changes with time, as shown in Figs 13–18.

Based on Figs 13–18, which show the shear stress-time history curves of the measured points, the following results can be summarized:

1. The shear stress of measuring point 1 is slightly larger than the value of measuring point 2 because measuring point 1 is close to the soil sample box; thus, the compressive stress is higher than that of measuring point 2, and under the action of the compressive stress, the shear stress will increase slightly.
2. From measuring point 2 to measuring point 4, the shear stress shows an upward trend; from measuring point 4 to measuring point 6, the shear stress shows a downward trend.
3. Conditioned soil begins to accumulate between measuring point 2 and measuring point 3, and the soil accumulation section is completed near measuring point 4. The soil accumulation section is directed to the outlet under the action of the helical casing shear stress. Under the unloading action of the muck outlet, the shear stress of the measuring point decreases.

**Fig 19:**
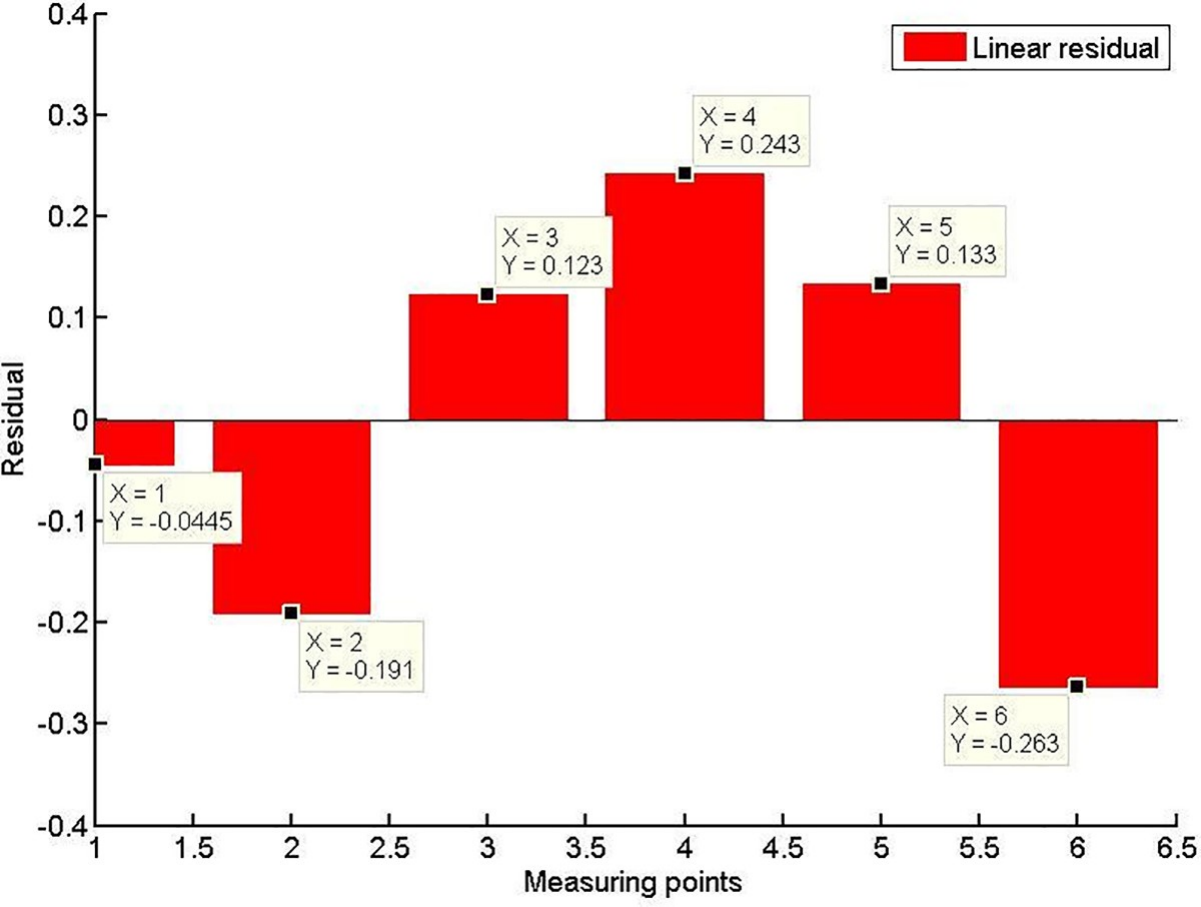
Fitting residuals at each measuring point.

When the model screw conveyor is working normally, the linear fitting residual of the shear stress at each measuring point indicates that the shear stress of the spiral casing is approximately constant, as shown in Fig 19. The mechanism of the change of spiral shear stress with compressive stress will be the next problem studied.

**Fig 20:**
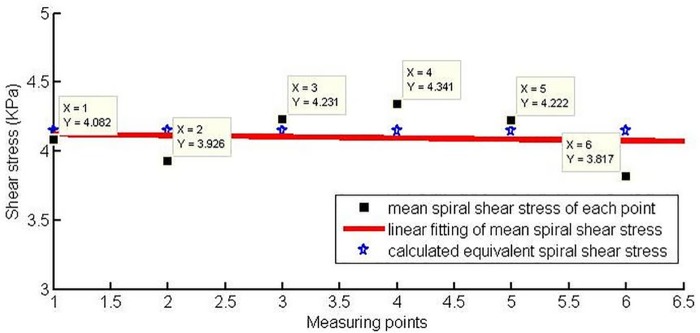
Comparison of the mean theoretical value and linear fitting value.

From the comparison results in Fig 20, the mean shear stress of each measuring point is close to the theoretical calculation result, that is, 4.15 *KPa*. It is shown that the equivalent spiral shear stress calculation model derived from the equilibrium condition of moment of momentum can accurately predict the shear behavior of the conditioned soil. Moreover, the calculation results are related to the physical and mechanical parameters of the conditioned muck, the operating conditions, and the geometric structure of the shield screw conveyor.

## Conclusions

It is seen that the driving torque of a screw conveyor is closely related with the shear stress created at the interface between the conveyor casing and the conditioned soil. The driving torque is easy to measure, whereas the shear stress is difficult to get due to its occurrence especially in the conveyor casing of a real shield machine. From the screw conveyor model machine tests, the shear stress along the casing is measured. The measured results coincide well the calculated results by Eq (23), which also validate the derived relationship between the driving torque and the shear stress.

Also, after the optimization scheme of the soil conditioning is determined, the indoor test of the screw conveyor model can be carried out with the conditioned soil. With the measured torque, the shear stress of the conditioned soil under the current improvement scheme and the pressurized condition is solved by Eq (23). Then the parameter of the shear stress is combined with the relevant parameters of the screw conveyor and the conditioning scheme, the influence of the soil improvement scheme on the performance of the shield tunneling can be evaluated by the Eq (23). Based on the above discussions, the following conclusions are drawn:

1. The driving torque of a screw conveyor is heavily dependent on the shear stress created at the interface between the conditioned soil and the conveyor casing. A linear relationship between them is derived in theory. The shear stress is also affected by the conveyor geometric parameters, the density of the conditioned muck and the installation angle of the conveyor. Using the derived equation, the shear stress can be quantitatively determined for given driving torque and vice versa.
2. Measured results from the indoor tests using the developed conveyor model machine confirmed the validity of the derived equation at least for the conditioned standard sand. As for other soils, such as clay soils, further tests are still needed in the future.
3. On the basis of the conveyor model machine test and using the derived equation, the shear stress at the interface between the muck and the conveyor casing can be more accurately modelled, which provides a better indicator of EPB shield tunneling performance by soil conditioning.
4. The indoor test performed using the screw conveyor model machine, there still exist limitations. In actual engineering, the temperatures of the conditioned soil range from 30 to 40°C or even higher. However, this factor is not taken into account during the indoor test. In addition, the soil conditioning is carried out simultaneously during the process of the tunnel excavation. Whereas, in the indoor test the conditioned muck is prepared first, and then poured into a sand sample box. The impact of these two different soil conditioning improvement processes on the performance of the shield screw conveyor needs further study. Moreover, the issues of wear and abrasion can't be included in the laboratory tests.

These conclusions provide some positive guidelines and will be helpful for optimizing conditioning sandy stratum and design a better EPB shield screw conveyor machine, especially one that operates in noncohesive soil. Our subsequent research will focus on other stress parameters of the screw conveyor regarding undrained shear strengths, as well as adapting the machine to different geological conditions.

## Supporting information

S1 Table List of notation. For the convenience of analysis, the symbols used in the paper are listed in S1 Table.
(DOCX)
