# Supplementary material for: Calculation model of the equivalent spiral shear stress of conditioned sand
Source: PLoS One. 2019 Mar 13;14(3):e0212923. doi: 10.1371/journal.pone.0212923 (PMC6415801; doi:10.1371/journal.pone.0212923)
Supplement: S1 Table — For the convenience of analysis, the symbols used in the paper are listed in S1 Table. (DOCX) [file pone.0212923.s001.docx]

For the convenience of analysis, the symbols used in the paperare listed in S1 Table.

**S1 Table. List of Notation.**

| **Symbol** | **Indicates** |
| --- | --- |
| *e* | The thickness of the spiral blade |
| *s* | The pitch |
| *2r* | The diameter of the spiral shaft |
| *2R* | The diameter of the spiral blade |
| *φ* | The helix angle |
| *y* | The spiral shaft direction |
| *x* | The vertical spiral shaft direction |
| *u* | The vertical spiral blade direction |
| *l* | The spiral balde direction |
| *dl* | The microincrement along the spiral blade direction |
| *dy* | The microincrement along the spiral shaftdirection |
| *d(f_t_)_l_* | The top length of the microbody along the spiral blade direction |
| *d(f_m_)_l_* | The average lengthof the microbody along the spiral blade direction |
| *d(f_b_)_l_* | The bottom lengthof the microbody along the spiral blade direction |
| (*c_t_*)*_w_* | The top width of the microbody in the groove |
| (*c_m_*)*_w_* | The average width ofthe microbody in the groove |
| (*c_b_*)*_w_* | The bottom widthof the microbody in the groove |
| *d* | The depth of the groove |
| *φ_m_* | The average helix angle of the blade |
| *φ_s_* | The helix angle at the spiral shaft |
| (*τ_f_*)*_t_* | The interface shear stress between the upper spiral blade and conditioned soil |
| (*τ_f_*)*_b_* | The interface shear stress between the lower spiral blade and conditioned soil |
| *τ_s_* | The interface shear stress between the spiralshaft and conditioned soil |
| *τ_c_* | The interface shear stress between the spiral shell and conditioned soil |
| *Q_n_* | The combined pressing force of the upper and lower spiral blades |
| *P* | The pressure in the direction of the spiral groove |
| *ΔP* | The pressure microincrement along the spiral groove direction |
| *G* | The weight of the soil microbody |
| *φ* | The slope when the model screw conveyor is installed |
| *θ* | The flow angle of the soil |
| *n* | The rotational speed of the spiral blade, rpm |
| *ρ* | The conditioned soil density |
| *g* | The acceleration of gravity |
| *π* | A mathematical constant, the ratio of a circle's circumference to its diameter, approximated as 3.14 |
| *τ_y_* | The vertical shear stress along the spiral shaft direction, *KPa* |
| *τ_x_* | The horizontal shear stress perpendicular to the spiral shaft direction, *KPa* |
| *τ_r_* | The synthesized shear stress of each measuring point,*KPa* |
